# Validation in *Drosophila* of the *in silico* predicted clomipramine as repurposable for SOD1-ALS

**DOI:** 10.1016/j.neurot.2025.e00793

**Published:** 2025-11-14

**Authors:** Francesco Liguori, Susanna Amadio, Chiara Angioli, Angelo Ferriero, Iolanda Passaro, Francesca Alberti, Fiammetta Vernì, Cinzia Volonté

**Affiliations:** a Experimental Neuroscience and Neurological Disease Models, IRCCS Fondazione Santa Lucia, Via del Fosso di Fiorano 65, 00143, Rome, Italy; b Institute for Systems Analysis and Computer Science “Antonio Ruberti” (IASI), National Research Council (CNR), Via dei Taurini 19, 00185, Rome, Italy; c Department of Biology and Biotechnology “Charles Darwin”, Sapienza University of Rome, Piazzale Aldo Moro 5, 00185, Rome, Italy

**Keywords:** *Drosophila*, Drug repurposing, Amyotrophic lateral sclerosis, Clomipramine, DNA damage, Inflammation

## Abstract

Amyotrophic lateral sclerosis (ALS) is a devastating neurodegenerative disease characterized by progressive motor neuron degeneration and muscle weakness, generally leading to death due to respiratory failure within 2–5 years of symptom onset. Current Food and Drug Administration-approved drugs —riluzole, edaravone, and tofersen — offer limited clinical benefit due to ALS multifactorial etiology and high heterogeneity. To bypass this therapeutic letdown, we previously exploited network medicine and drug repurposing strategies. Leveraging the SAveRUNNER algorithm, we identified several potentially repurposable candidates, including clomipramine (Anafranil®), mianserin (Lantanon®/Tolvon®), and modafinil (Provigil®). Here, we evaluated the *in vivo* efficacy of these compounds in *Drosophila* models of ALS, precisely those expressing pan-neuronal human *SOD1*^*A4V*^ or *SOD1*^*G85R*^ mutations. Our results demonstrate that clomipramine is the most promising candidate, ameliorating lifespan reduction, improving climbing abilities, and mitigating both genomic instability and inflammation, key pathological hallmarks of these SOD1-ALS models. Despite needing further validation in higher organisms, our *Drosophila* findings represent preliminary yet significant support for clomipramine's action as an add-on treatment for SOD1-ALS.

## Graphical abstract

**Figure:**
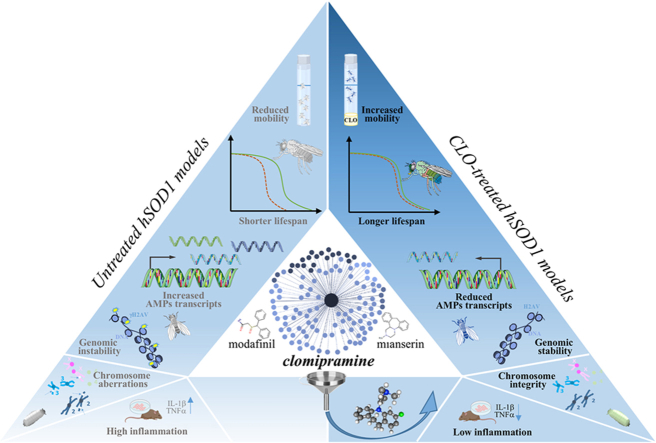

## Introduction

Amyotrophic lateral sclerosis (ALS) (OMIM #105400) is a fatal neurodegenerative disease characterized by progressive motor neuron degeneration and muscle weakness, culminating in respiratory failure and paralysis that limit patient survival to 2–5 years after the onset of symptoms. In about 50 ​% of cases, extra-motor manifestations also occur, such as behavioral changes, dysphagia, and speech disorders [[1], [2], [3]]. ALS has an incidence of ∼2 cases per 100.000 people per year. Despite its rarity, we estimate that, due to population aging, the number of ALS cases worldwide is expected to continuously increase [4,5]. However, it's important to note that these data underestimate the true frequency of ALS, because current incidence rates exclude undeveloped non-industrial countries and are moreover based only on patients who have already developed symptoms: this means that ALS presymptomatic individuals are excluded, leading to an incomplete picture of the actual prevalence. This gap and the ever-increasing incidence, thus testify to the urgent need for new therapeutics against this multi-systemic disease that, to date, is still incurable.

The current drugs approved by U.S. Food and Drug Administration for the treatment of ALS are: i) riluzole (Rilutek®/Teglutik®/Exservan®), approved back in 1995 [[6], [7], [8]] that inhibits glutamate excitotoxicity and excessive motor neuron firing, ii) edaravone (Radicava®), a free radical scavenger distributed since 2017 that reduces oxidative stress [9], and iii) the recently-approved tofersen (Qalsody®) [10], an antisense oligonucleotide mRNA targeting *Superoxide Dismutase 1* (*SOD1 –* the second most frequent ALS-causative gene) [1] that improves respiratory failure and general quality of life. However, despite impressive research and clinical efforts worldwide, none of these compounds offers significant clinical benefit [11,12]. The reasons for this therapeutic failure are multiple and include: i) ALS complex and multifactorial etiology with the involvement over time of both genetic and environmental factors, ii) multiple cellular mechanisms determining motor neuron degeneration (e.g., inflammation, excitotoxicity, oxidative stress, and mitochondrial dysfunction), and iii) extreme heterogeneity in terms of age of onset, rate of progression, pattern of inheritance, and symptomatology [13]. In this composite scenario, we do consider network medicine and drug repurposing as particularly promising approaches.

Based on these premises, we previously adopted the algorithm SAveRUNNER (Searching off-lAbel dRUg aNd NEtwoRk) [14] to identify some existing drugs as repurposable candidates against ALS [15]. Within the 121 molecules identified by SAveRUNNER as ALS-associated according to stringent predictive features, our focus was on 3-(3-chloro-10,11-dihydro-5H-dibenzo[b,f]azepin-5-yl)-N,N-dimethylpropan-1-amine (clomipramine, Anafranil®), the dibenzazepine-derivative and tricyclic 3-chloro tertiary amine analog of imipramine. Clomipramine is mainly in use for the treatment of obsessive-compulsive disorders [16] and other mood disorders such as depression, schizophrenia, and Tourette's syndrome, thanks to its action as a norepinephrine and serotonin reuptake inhibitor [17,18]. However, beyond this primary inhibitory action, clomipramine also affects other neurotransmitter systems by blocking histamine, dopamine, and acetylcholine receptors (Table 1). Interestingly, recent studies in murine models report that clomipramine also improves the symptoms of neurological and inflammatory diseases such as chronic and acute phases of multiple sclerosis [19] and ulcerative colitis [20]. Indeed, its ability to act on multiple molecular targets was considered a highly beneficial element against a complex disease like ALS, which necessitates of broad-spectrum therapeutic interventions [21].

**Table 1:** **Pharmacological profile of clomipramine.** The table summarizes some key pharmacological characteristics of clomipramine (Drug Bank ID: DB01242), including its chemical structure, established clinical indications, off label uses and primary mechanisms of action.

Structure	Indications	Off label uses	Mechanisms of action
	- Obsessive disorder - Depression - Schizophrenia - Tourette's syndrome	- Chronic pain - Panic disorder - Cataplexy - Narcolepsy - Premature ejaculation - Premenstrual syndrome	**Reuptake inhibitor:** - Serotonin - Noradrenaline **Antagonist:** - 5-HT2A, 5-HT2B, 5-HT2C, 5-HT3, 5-HT6, 5-HT7 serotonin receptors - M1, M2, M3 muscarinic receptors - D1, D2, D3 dopamine receptors - H1 histaminergic receptors - α1 adrenergic receptors

The aim of the present work was therefore to evaluate *in vivo* the efficacy of clomipramine in modulating some ALS hallmarks using our recently characterized *Drosophila* models pan-neuronally expressing *hSOD1*^*A4V*^ or *hSOD1*^*G85R*^ ALS-associated human mutations [22].

## Materials and methods

### Fly stocks

*Drosophila* stocks were obtained from Bloomington *Drosophila* Stock Center (Bloomington, IN, USA) and are:

- w^1118^; P-w[+mC]=UAS-hSOD1.A4V-9.1/TM6B, Tb^1^ (BL33607)
- w^1118^; P-w[+mC]=UAS-hSOD1.G85R-2a; 2b; 3a; 3b (BL33608)
- P-w[+mW.hs]=GawB-elav[C155] (BL458)

*OregonR* wild type strain and balancer stocks are part of the laboratory's collection. Transgenic flies were backcrossed to the laboratory *OregonR* strain for at least six generations to produce isogenic lines. Stocks were maintained at 25 ​°C, 70 ​% humidity, in a 12:12 light/dark cycle (light on at 9:00 a.m., light off at 9:00 p.m.) and fed with standard sugar-cornmeal-yeast-agar nutritional medium, supplemented with 0.75 ​% propionic acid (cat. 409553, Carlo Erba Reagents, Cornaredo, Italy) as mold inhibitor. All genetic crosses were performed at 25 ​°C.

### Drug treatments

Clomipramine Hydrochloride (cat. C7291, Merck Life Science, Milan, Italy) and Mianserin Hydrochloride (cat. M2525, Merck Life Science) were dissolved into sterile water to obtain 356 ​mM and 91 ​mM stock solutions, respectively, and then diluted into fly nutritional medium at 0.5 ​mM final concentration. Modafinil (cat. Y0000635, Merck Life Science) was dissolved into DMSO to obtain 11 ​mM stock solution and then diluted into fly nutritional medium at 0.05 ​mM final concentration.

For lifespan and climbing assays, adult flies were chronically drug-treated since their emergence from pupal case (eclosion) with transfer to fresh vials every 3 days. Flies used to perform other experimental procedures (DNA, RNA and protein extractions, AChE enzymatic activity measurement) were drug-treated for 17–20 days and then their heads were isolated. We selected the timepoint 17–20 days in order to guarantee a condition of full survival, since some *hSOD1*^*A4V*^ and *hSOD1*^*G85R*^ flies start to die just after 20 days. Age-matched controls were reared on standard medium (for clomipramine and mianserin treatments) or DMSO-supplemented medium (for modafinil treatments). Third-instar *hSOD1*-expressing larvae were obtained by crossing parental lines on drug-supplemented medium (or vehicle) to ensure that F1 generation was exposed to the drug (or vehicle) for the entire developmental period. To determine the optimal dose for each compound, survival assays were conducted on 50 *OregonR* wild-type flies reared on standard food medium supplemented with increasing drug concentrations. Flies were monitored daily to establish the tolerability dose-response curve. The chosen optimal dose for subsequent experiments was the one causing less than 15 ​% loss of median lifespan respect to vehicle (see Supp. Figg. 1, 3 and 4).

### Lifespan assay

For each genotype and experimental group, the entire cohort of flies was divided into different vials, each containing 20 individuals. Every other day, flies were transferred to fresh vials, and mortality was recorded. Flies that escaped or were accidently lost were considered right-censored and excluded from survival analysis.

### Climbing assay

For each genotype and experimental condition, flies were divided into vials, each containing 10 individuals. Negative geotaxis assays were performed at 3–6, 9–12, 17–20, 25–28, and 33–36 days post-eclosion. Briefly, flies were transferred to empty vials marked with a line at 8 ​cm from the base. Vials were gently tapped to induce a negative geotaxic response. The number of flies crossing the 8 ​cm line within 10 ​s was recorded. Each vial was tested in triplicate, and the mean number of flies reaching the target line, expressed as mean ​± ​SEM, was calculated and graphically represented. To minimize variability due to circadian rhythm, all assays were consistently performed at the same time during the day. To avoid the known confounding effects of the dark phase and light-dark transitions on *Drosophila* locomotor activity, climbing assays were performed around 3:00 p.m. (ZT6), approximately midway through the light phase.

### Determination of acetylcholinesterase (AChE) activity

AChE activity in *Drosophila* heads was assessed according to Ref. [23]. Briefly, 15 heads isolated from 20-days-old flies/genotype/condition were homogenized in 0.1 ​M phosphate buffer pH 7.4 and then centrifuged at 10.000 ​rpm for 15 ​min at 4 ​°C. Supernatants were diluted in a ratio 1:5 with 10 ​mM phosphate buffer pH 7.4 and then added to a reaction mixture made by 1 ​mM Ellman's reagent (cat. 22582, Thermo Fisher Scientific, Waltham, MA, USA), 0.8 ​mM acetylthiocholine (cat. S01480, Merck Life Science) and 1 ​mM phosphate buffer pH 7.4. Samples were then assayed spectrophotometrically at 412 ​nm over a period of 5 ​min at 15-s intervals. Obtained OD values were averaged and used to calculate AChE enzymatic activity, expressed in μmol/mL/min. AChE activity was repeated with three different fly crosses.

### Total RNA extraction, reverse transcription and qRT-PCR

30 heads/genotype/condition were harvested and stored at −20 ​°C in RNA later (cat. R0901, Sigma Aldrich, Darmstadt, Germania). RNA was isolated by QIAzol lysis reagent (cat. 79306, QIAgen, Hilden, Germany) according to the manufacturer's protocol. RNA samples were quality- and quantity-checked and then reverse-transcribed using PrimeScript RT Reagent Kit with gDNA Eraser (cat. RR047A, Takara Bio, Kyoto, Japan). 30 ​ng of cDNA were used to carry out qRT-PCR reactions with TB Green Premix Ex Taq II (cat. RR420W, Takara Bio) applying the following thermal profile: initial denaturation at 95 ​°C for 10 ​min, followed by 35 cycles of 10 ​s at 95 ​°C and 30 ​s at 60 ​°C. Melting curves were obtained after 10 ​s at 95 ​°C and 1 ​min at 65 ​°C. At least three independent biological replicates were performed with three technical replicates each. Relative quantification of the transcripts was determined by the 2^–ΔΔCt^ method [24] with *actin* used as reference housekeeping gene. The sequences of primers used (SIAL, Rome, Italy) were as follows:

• Dm_Actin_Forward: GCTTCGCTGTCTACTTTCCA
• Dm_Actin_Reverse: CAGCCCGACTACTGCTTAGA
• Dm_Attacin_Forward: GGGCTACAACAATCATGGA
• Dm_Attacin_Reverse: GACCTTTGATGTGGGAGTAG
• Dm_Cecropin_Forward: AAGCTGGGTGGCTGAAGAAA
• Dm_Cecropin_Reverse: TGTTGAGCGATTCCCAGTCC
• Dm_Diptericin_Reverse: ATCCTTGCTTTGGGCTTC
• Dm_Diptericin_Forward: CCTGAACCACTGGCATAT
• Dm_Drosocin_Forward: TTTTCCTGCTGCTTGCTTGC
• Dm_Drosocin_Reverse: GGCAGCTTGAGTCAGGTGAT

### Mitotic chromosome preparation

To assess metaphase chromosome aberrations, colchicine-treated brains isolated from third instar larvae fully developed on a nutritional drug-supplemented medium (and correspondent untreated controls) were processed as described in Ref. [25]. Fixed samples were then mounted in Vectashield H-1200 with DAPI (Vector Laboratories, Newark, CA, USA) for DNA staining. The resulting cytological preparations were analyzed using a Carl Zeiss Axioplan fluorescence microscope, equipped with HBO100W mercury lamp and cooled charged-coupled device (CCD camera; Photometrics CoolSnap HQ). A minimum of 500 ​cells per condition were evaluated across 5 to 10 brains.

### Immunostaining and γH2AV foci detection

Third-instar larval brains of each genotype/condition were dissected in PBS and fixed in 4 ​% formaldehyde PBS for 20 ​min. Samples were then rinsed in 45 ​% acetic acid and kept in 60 ​% acetic acid for 2 ​min onto a glass coverslip. Brains were then squashed and fragmented by a glass slide, frozen in liquid nitrogen, washed twice in PBS 0.1 ​% Triton (PBST), and incubated overnight at 4 ​°C with rabbit anti-Histone H2AvD pS137 (γH2AV) primary antibody (1:100 in PBST; cat. 600-401-914, Rockland Immunochemicals, Limerick, PA, USA). The next day, brains were rinsed twice in PBST and incubated for 1 ​h at room temperature with donkey anti-rabbit Alexa Fluor 488-conjugate (1:300 in PBST). After two additional PBST washes, slides were mounted in Fluoromount-G with DAPI (cat. 00495952, Thermo Fisher Scientific). All cytological preparations were examined using a confocal laser scanning microscope (LSM800, Zeiss) equipped with 405 ​nm, 488 ​nm, 561 ​nm, and 639 ​nm laser sources. Digital image processing, including brightness and contrast adjustments, was conducted using Zeiss Zen software 3.0 blue edition (Zeiss) and Adobe Photoshop (Adobe, USA). To quantify γH2AV foci, at least 2000 ​cells per genotype/condition were analyzed by direct counting, thus obtaining the foci/total nuclei ratio.

### Western blotting

Total protein extracts were prepared by homogenizing 10 fly heads in RIPA buffer supplemented with 10 ​mM NaF and 1 ​mM Na_3_VO_4_ as phosphatase inhibitors. Supernatants were then heated at 85 ​°C for 8 ​min. Proteins were separated by SDS-PAGE and transferred onto nitrocellulose membranes (cat. GE10600002, Protran, Merck, Darmstadt, Germany) in 20 ​% ethanol in Tris-Glycine buffer. Membranes were blocked for 30 ​min in 5 ​% non-fat dry milk TBS-T (10 ​mM Tris pH 8, 150 ​mM NaCl, 0.1 ​% Tween 20) and then incubated overnight at 4 ​°C with the indicated primary antibodies. Membranes were subsequently incubated with HRP-conjugated anti-rabbit secondary antibody (1:5000 in TBS-T buffer) for 1 ​h at room temperature. The conserved and ubiquitous Giotto protein served as loading control. Immunostained bands were visualized using Super Signal West Pico Plus ECL (Thermo Scientific) on iBright CL1000 Imaging System (Invitrogen, Waltham, MA, USA) and quantified using ImageJ software.

Primary antibodies:

• rabbit anti-H2AvD pS137 1:500 - Rockland Immunochemicals
• rabbit anti-AChE 1:5000 – [26].
• rabbit anti-giotto 1:10000 – [27].

### Genomic DNA extraction and long amplicon (LA)-PCR

Genomic DNA was isolated from 30 heads per genotype/treatment using Cell and Tissue DNA Isolation Kit (Norgen Biotek Corp., Thorold, Canada) according to manufacturer's protocol. DNA was then quantity- and quality-checked, and 20 ​ng were used as template for LA-PCR with the TaKaRa LA Taq Kit (cat. RR002 ​M, Takara Bio). The LA-PCR thermal cycling conditions were based on [28]: initial denaturation at 94 ​°C for 30 ​s, followed by 25 cycles of denaturation (94 ​°C for 30 ​s), annealing (60 ​°C for 30 ​s), and extension (65 ​°C for 10 ​min), with a final extension at 65 ​°C for 10 ​min. The *Neurexin* gene was amplified, with a large region (LA) targeted to assess DNA damage and a smaller region (SA) for normalization. The PCR products were visualized by gel electrophoresis and quantified using ImageJ software. Primers used were purchased from SIAL and were:

• Dm_Neurexin_LA_Forward: GGTACACCGGACTGAATGGG
• Dm_Neurexin_LA_Reverse: CTGGCTACACTTGGTGGGTC
• Dm_Neurexin_SA_Forward: TGCAACAACAGTGCCCTACT
• Dm_Neurexin_SA_Reverse: TAGCCTTAACGAGCGACCAC

### NSC-34 ​cell line

The NSC-34 ​cell clone (CVCL_D356) used for our experiments [29] was a kind gift by Prof. Fabrizio Chiti (University of Florence, Italy). Cell culture conditions, RNA extraction and qPCR were as reported in Ref. [30]. NSC-34 ​cells were transiently transfected with human *SOD1* cDNA containing the G93A mutation, cloned into the pRc/CMV plasmid (a kind gift by Dr. Alberto Ferri, IFT-CNR, Rome, Italy). Control cells were transfected with the empty pRc/CMV plasmid (lacking the *SOD1* gene). Cells (100.000/well) were plated in a 12-well multiwell plate. After 24 ​h, at approximately 80 ​% confluence, cells were treated with jetOPTIMUS DNA Transfection Reagent (Polyplus, Illkirch, France) according to the manufacturer's protocol. Following additional 24 ​h, the culture medium was replaced, and cells were treated with 1 ​μM or 10 ​μM clomipramine [31].

The sequences of primers used for qPCR (SIAL) were as follows:

• Mm_IL-1β_Forward: TGGACCTTCCAGGATGAGGACA
• Mm_IL-1β_Reverse: GTTCATCTCGGAGCCTGTAGTG
• Mm_TNFα_Forward: GGTGCCTATGTCTCAGCCTCTT
• Mm_TNFα_Reverse: GCCATAGAACTGATGAGAGGGAG
• Mm_TATA box BP_Forward: CCAATGACTCCTATGACCCCTA
• Mm_TATA box BP_Reverse: CAGCCAAGATTCACGGTAGAT

### Statistical analysis

The distribution of the data was tested for normality through Shapiro-Wilk test. Lifespan curves significance was assessed through log-rank test with Bonferroni's correction. Climbing assays were analyzed with three-way mixed design Analysis of Variance (ANOVA), with time as the repeated measures factor, followed by Tukey's *post hoc* test for multiple comparisons. To specifically test the differential effect of clomipramine, Delta (Δ) analysis (Treated *minus* Untreated) was performed on the climbing data, followed by a two-way ANOVA with Sidak's *post hoc* test to evaluate the genotype ​× ​time interaction on the drug-induced performance change. The statistical significance of AChE enzymatic activity, western blots for AChE and γH2AV, CABs frequency, γH2AV immunofluorescence and LA-PCR was assessed through two-way ANOVA followed by Sidak's *post hoc* test for multiple comparisons. For qPCR results analysis two-way ANOVA with Dunnet's *post hoc* test was used. All data were analyzed through GraphPad Prism 8.0 software, exception made for lifespan experiments, whose curves were generated and analyzed with OASIS2 software (https://sbi.postech.ac.kr/oasis2) [32].

## Results

### Clomipramine ameliorates lifespan in SOD1-ALS flies

Our previous work established that pan-neuronal expression of human ALS-associated *SOD1*^*A4V*^ and *SOD1*^*G85R*^ mutations in *Drosophila* leads to a significant reduction of lifespan when compared to non-mutant controls [22]. In this study we performed different assays to verify whether clomipramine mitigates the phenotypes observed in *elav-Gal4>UAS-hSOD1*^*A4V*^ and *elav-Gal4>UAS-hSOD1*^*G85R*^ fly models (respectively named *hSOD1*^*A4V*^ and *hSOD1*^*G85R*^ from now on).

**Fig. 1:**
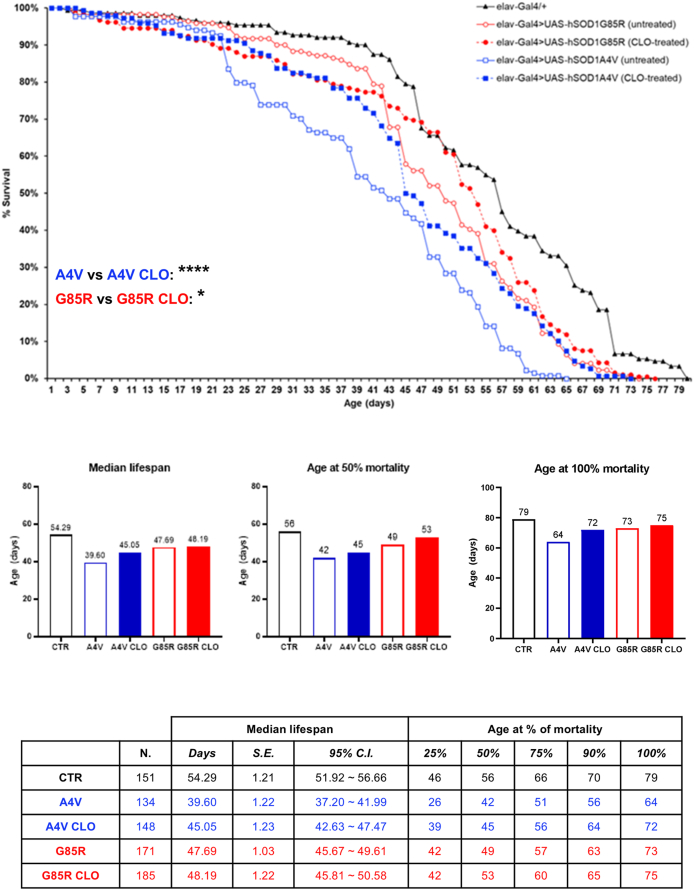
**Clomipramine modulates survival in *hSOD1*^*A4V*^ and *hSOD1*^*G85R*^ ALS flies**. Survival curves of: *elav-Gal4/+* (CTR, black line, black triangles, n ​= ​151), *elav-Gal4>UAS-hSOD1*^*A4V*^ untreated (A4V, blue line, empty blue squares, n ​= ​134) and clomipramine-treated (A4V CLO, blue line, full blue squares, n ​= ​148), *elav-Gal4>hSOD1*^*G85R*^ untreated (G85R, red line, empty red circles, n ​= ​171) and clomipramine-treated (G85R CLO, red line, full red circles, n ​= ​185). Data are pooled from three independent biological replicates, each originating from separate parental crosses. Statistical significance was calculated by Log-rank test with Bonferroni correction through OASIS2 online software, A4V vs A4V CLO: ∗∗∗∗p ​< ​0.0001, G85R vs G85R CLO: ∗p ​< ​0.05. Bar graphs from left to right show the difference in terms of median lifespan, age at 50 ​% and at 100 ​% survival, respectively. The table summarizes the total number of flies analyzed in clomipramine-treated survival curves and their respective controls, median lifespan ​± ​S.E. (standard error) with 95 ​% C.I. (confidence interval), and fly age (in days) at 25 ​%, 50 ​%, 75 ​%, 90 ​%, and 100 ​% mortality for each genotype.

**Fig. S1:**
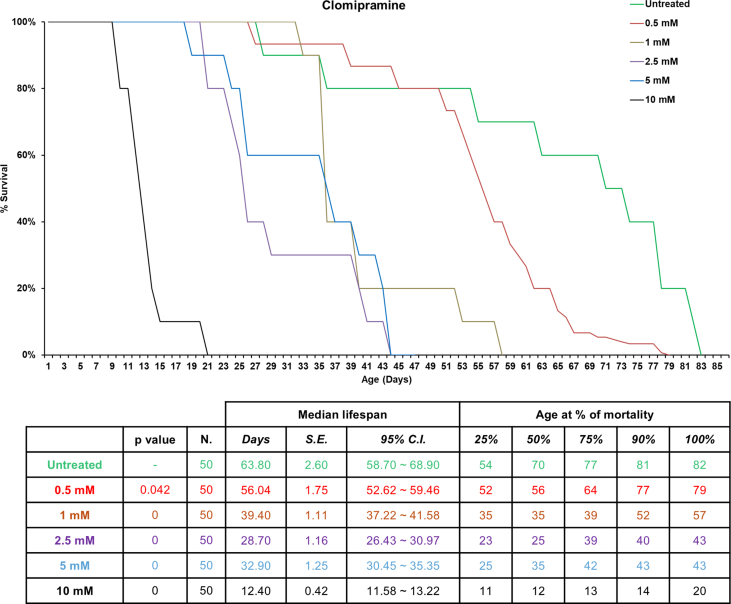
**Survival curves of wild type flies to determine the optimal dose of clomipramine.** Survival curves of 50 wild type *OregonR* flies administered with mounting doses of clomipramine (0.5 ​mM – red line; 1 ​mM – brown line; 2.5 ​mM purple line; 5 ​mM – blue line; 10 ​mM black line). Green line indicates the survival of the flies reared on normal medium. 0.5 ​mM clomipramine was selected for further experiments as the optimal dose because it induced less than 15 ​% loss of median lifespan with respect to the vehicle. The log-rank test with Bonferroni's correction established that 0.5 ​mM induced a minimal, yet statistically significant, reduction in *OregonR* lifespan compared to the vehicle (p ​= ​0.042). This effect was further supported by the Wilcoxon-Breslow-Gehan test (p ​= ​0.0145), in turn establishing that the observed toxicity was due to early-phase death. All higher concentrations induced acute toxicity (p ≈ 0 for all comparisons). The table summarizes the number of flies analyzed, the Bonferroni p value, their median lifespan ​± ​S.E. (standard error) with 95 ​% C.I. (confidence interval), and fly age (in days) at 25 ​%, 50 ​%, 75 ​%, 90 ​%, and 100 ​% mortality for each tested concentration.

After establishing the optimal dose of 0.5 ​mM based on tolerability assays (Supp. Fig. 1), we evaluated clomipramine's effect on lifespan. Clomipramine induced a significant increase of 12.3 ​% in lifespan of *hSOD1*^*A4V*^ mutant flies and a more modest, yet still statistically significant improvement of 2.8 ​% in *hSOD1*^*G85R*^ mutant flies (Fig. 1). This marked difference in efficacy suggests a mutation-specific response to the drug.

### Clomipramine modulates motor abilities in a mutation-dependent manner

**Fig. 2:**
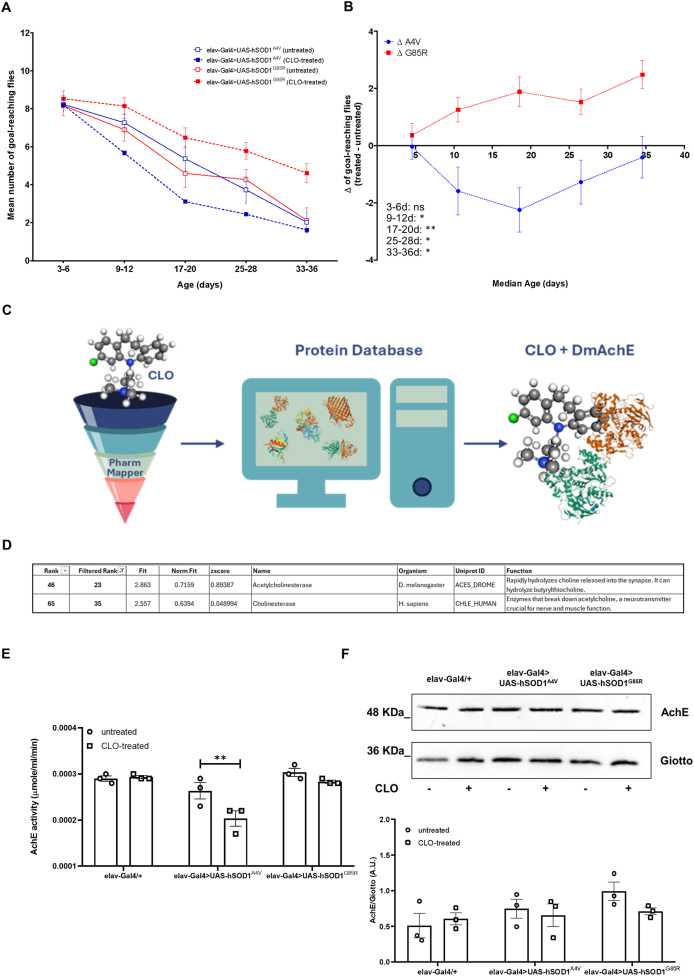
**Clomipramine modulates locomotor activity of *hSOD1*-ALS flies in a mutation-dependent manner. A)** Negative geotaxis assay at different time-points (3–6, 9–12, 17–20, 25–28 and 33–36 days post-eclosion) of untreated (blue line, empty blue squares) and treated (blue line, full blue squares) *elav-Gal4>UAS-hSOD1*^*A4V*^ flies and untreated (red line, empty red circles) and treated (red line, full red circles) *elav-Gal4>UAS-hSOD1*^*G85R*^ flies. At least 100 flies (pooled from three separate parental crosses) for each genotype/treatment were analyzed. Locomotor ability is represented as the mean number of flies reaching the goal of 8 ​cm from the bottom of the vial within 10 ​s ​± ​SEM. Each vial was tested three times. Statistical significance was calculated by three-way mixed-design ANOVA (time as repeated factor) followed by Tukey's *post hoc* test for multiple comparisons. The overall ANOVA revealed significant effects for time (F_(2.97, 107.0)_ ​= ​102.0, p ​< ​0.0001), genotype (F_(1,36)_ ​= ​7.091, p ​= ​0.0115) and a highly significant genotype ​× ​treatment interaction (F_(1,36)_ ​= ​8.515, p ​= ​0.0060). No other interactions were significant (time ​× ​genotype ​× ​treatment, p ​= ​0.0648). Tukey's *post hoc* test did not detect significance in the direct comparisons between treated and untreated groups at corresponding time-points. Therefore, to specifically validate the mutation-dependent and opposite effects of clomipramine, Delta (Δ) analysis followed by two-way ANOVA was performed (see Fig. 2B). **B)** Delta (Δ) analysis on locomotor performance of untreated and treated *elav-Gal4>UAS-hSOD1*^*A4V*^ and *elav-Gal4>UAS-hSOD1*^*G85R*^ flies. The Δ values were calculated as the difference between the performance of treated and untreated flies (Δ ​= ​treated *minus* untreated) and illustrate the effect of clomipramine on the two *hSOD1* mutants over time. Positive Δ values indicate functional improvement, while negative Δ values indicate functional decline compared to the untreated control. Statistical analysis was performed using two-way ANOVA (time as repeated factor, genotype as between factor) on the Δ values. This analysis revealed a highly significant main effect of genotype (F [1,18] ​= ​17.75, p ​= ​0.0005). Furthermore, a significant genotype ​× ​time interaction was found (F [4,72] ​= ​4.195, p ​= ​0.0041). Sidak's multiple comparisons *post hoc* test was performed to compare Δ A4V with Δ G85R at each time-point. The analysis showed a significant difference between the two mutants at 9−12 (∗p ​= ​0.0472), 17−20 (∗∗p ​= ​0.0022), at 25−28 (∗p ​= ​0.0334) and at 33−36 days (∗p ​= ​0.0231). Data are presented as mean ​± ​SEM. **C)** Workflow of PharmMapper (www.lilab-ecust.cn/pharmmapper): given the 3D structure of the query molecule CLO – clomipramine, the potential drug targets are searched into the several pharmacophore databases. **D)** The table reports the ranked list of clomipramine hit target of interest, sorted by fit score. **E)** Acetylcholinesterase (AChE) activity was measured in untreated (circles) and clomipramine-treated (squares) *elav-Gal4/+*, *elav-Gal4>UAS-hSOD1*^*A4V*^ and *elav-Gal4>UAS-hSOD1*^*G85R*^ adult fly heads. The results are expressed as mean ​± ​SEM of three independent experiments (as indicated by individual data points). Two-way ANOVA revealed a significant main effect of the genotype (F_(2, 12)_ ​= ​19.40, p ​= ​0.0002), of the treatment (F_(1, 12)_ ​= ​8.138, p ​= ​0.0145) and an interaction genotype x treatment (F_(2, 12)_ ​= ​4.262, p ​= ​0.04). Sidak's multiple comparisons test showed that clomipramine significantly reduced AChE activity only in the *hSOD1*^*A4V*^ group (∗∗p ​< ​0.01). **F)** Western blot analysis of *Drosophila* AChE expression in untreated and treated non-mutant (*elav-Gal4/+*) and ALS (*elav-Gal4>UAS-hSOD1*^*A4V*^ and *elav-Gal4>UAS-hSOD1*^*G85R*^) adult fly heads. The conserved and ubiquitous protein Giotto was used as loading control. Bar graphs represent the mean ​± ​SEM of the normalized ratio between AChE and Giotto obtained by three independent biological replicates, as represented by individual data points. Two-way ANOVA revealed no significant main effect of genotype (F_(2, 12)_ ​= ​2.608, p ​= ​0.1147), treatment (F_(1, 12)_ ​= ​0.7778, p ​= ​0.3952), or interaction (F_(2, 12)_ ​= ​1.088, p ​= ​0.3679) on AChE expression. Sidak's multiple comparisons test showed no statistically significant differences between untreated and clomipramine-treated flies for any of the tested genotypes.

**Fig. S2:**
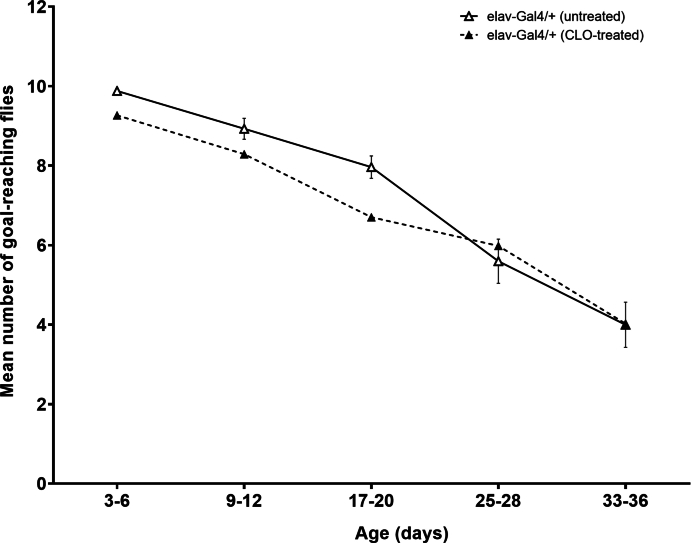
**Climbing performance of untreated and clomipramine-treated non-mutant flies.** Negative geotaxis assay at different time-points (3–6, 9–12, 17–20, 25–28 and 33–36 days post-eclosion) of untreated (black line, empty black triangles) and clomipramine-treated (black line, full black triangles) *elav-Gal4/+* flies. At least 100 flies (pooled from three separate parental crosses) for each condition were analyzed. Locomotor ability is represented as the mean number of flies reaching the goal of 8 ​cm from the bottom of the vial within 10 ​s ​± ​SEM. Each vial was tested three times. Statistical significance was calculated using a Two-way repeated measures ANOVA (time as the repeated factor) followed by Sidak's multiple comparisons test for *post hoc* analysis. The overall ANOVA revealed a highly significant main effect for time (F(_3.075, 55.35)_ ​= ​75.23, p ​< ​0.0001), indicating that motor activity significantly changed over the experimental period across both groups. No significant main effect was found for treatment (F_(1, 18)_ ​= ​1.324, p ​= ​0.2649), nor time ​× ​treatment interaction (F_(4, 72)_ ​= ​1.565, p ​= ​0.1928). Clomipramine treatment did not significantly alter the motor activity compared to the untreated control group. Sidak's *post hoc* test for direct comparisons at corresponding time-points confirmed the lack of significant differences across all individual time-points.

In order to comprehensively assess the therapeutic potential of clomipramine on mutant hSOD1-induced motor deficit, climbing assays were performed on *hSOD1*^*A4V*^ and *hSOD1*^*G85R*^ flies. Fig. 2A reports the motor performance data for all experimental conditions (both genotypes, untreated vs treated). This analysis revealed a significant interaction between genotype and treatment (p ​= ​0.006, by three-way ANOVA), thus demonstrating that the impact of 0.5 ​mM clomipramine on motor performance was mutation-dependent (clomipramine does not affect motor abilities of non-mutant controls *elav-Gal4/+* - Supp. Fig. 2). Specifically, in treated *hSOD1*^*A4V*^ flies an unexpected motor decline was observed. In contrast, treated *hSOD1*^*G85R*^ flies showed significantly enhanced motor performance across all age windows, compared to their untreated counterparts. This significant divergence in drug response is more clearly visualized by the Delta (Δ) analysis shown in Fig. 2B. In detail, the A4V Δ curve consistently drops below the zero line (thus indicating drug-induced toxicity), whereas the G85R Δ curve remains largely positive (indicating a drug-induced benefit).

To learn more about clomipramine's binding targets, we leveraged PharmMapper (www.lilab-ecust.cn/pharmmapper), a ligand-based pharmacophore mapping tool, designed to predict drug targets [33,34]. Based on the PharmMapper fit score, i.e. the rank that indicates how well the query molecule clomipramine fits to the target pharmacophore model (Fig. 2C), about 300 output ligands were obtained as potential clomipramine interactors (Supp. Table 1). The first within the *Drosophila* proteome was acetylcholinesterase (AChE), ranked at position #23 after filtering for and excluding outputs belonging to plants, prokaryotic organisms, and viruses (or position #46 without filtering). Interestingly, the human cholinesterase was found at position #35 (or #65 without filtering) in the PharmMapper output (Fig. 2D). Based on this predicted interaction, we directly measured AChE enzymatic activity and expression rate in our untreated and 0.5 ​mM clomipramine-treated fly heads. As reported in Fig. 2E, AChE enzymatic activity was significantly reduced by 23 ​% only in treated *hSOD1*^*A4V*^ flies respect to their untreated counterpart, notably without a corresponding change in total AChE protein levels (Fig. 2F). We do not exclude that inhibition of AChE in treated *hSOD1*^*A4V*^ fly heads might perhaps be correlated in some way to the motor ability decline caused by clomipramine only in these mutant flies.

### Clomipramine exerts anti-inflammatory action on SOD1-ALS flies and NSC-34 motor neuron-like cultures

**Fig. 3:**
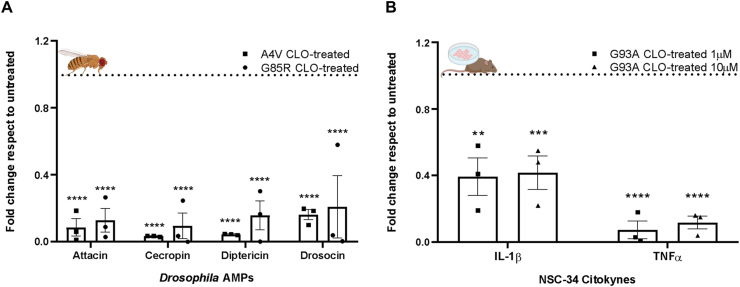
**Clomipramine reduces inflammation markers in *hSOD1*-ALS *in vivo* and *in vitro* models. A)** qPCR analysis of AMPs transcripts (attacin, cecropin, diptericin and drosocin) in *elav-Gal4>UAS-hSOD1*^*A4V*^ (squares) and *elav-Gal4>hSOD1*^*G85R*^ (circles) clomipramine-treated adult fly heads. Transcript levels were normalized to *actin* gene and are represented as fold change relative to each untreated genotype-matched sample (dotted horizontal line set to 1). Bar graphs represent the mean ​± ​SEM of three independent biological replicates and each with three technical replicates, as indicated by individual data points. Two-way ANOVA revealed a significant effect of clomipramine treatment (F_(2, 24)_ ​= ​221.7, p ​< ​0.0001), while there was no significant main effect of the AMP gene (F_(3, 24)_ ​= ​0.7256, p ​= ​0.5467) or interaction AMP gene x treatment (F_(6, 24)_ ​= ​0.2359, p ​= ​0.9604). Dunnett's multiple comparisons test showed that clomipramine significantly reduced the transcript levels of all four AMP genes (attacin, cecropin, diptericin, and drosocin) in both *elav−Gal4>UAS−hSOD1*^*A4V*^ and *elav−Gal4>hSOD1*^*G85R*^ flies, compared to their respective untreated controls (∗∗∗∗p ​< ​0.0001 for all comparisons). **B)** qPCR analysis of interleukin 1β (IL-1β) and tumor necrosis factor α (TNFα) transcripts in clomipramine-treated *hSOD1*^*G93A*^-NSC34 murine motor neuron-like cell line (clomipramine 1 ​μM – squares – and 10 ​μM – triangles –). Transcript levels were normalized to *TATA box binding protein* gene and represented as fold change relative to untreated cells. Bar graphs show the mean ​± ​SEM of at least three independent biological replicates and each with three technical replicates, as represented by individual data points. Two-way ANOVA revealed a significant main effect of treatment (F_(2, 12)_ ​= ​82.91, p ​< ​0.0001) and of cytokine (F_(1, 12)_ ​= ​14.14, p ​< ​0.01). No significant interaction cytokine x treatment (F_(2, 12)_ ​= ​3.547, p ​= ​0.0616) was observed. Dunnett's multiple comparisons test showed that clomipramine significantly reduced the transcript levels of IL-1β at both 1 ​μM (∗∗p ​< ​0.01) and 10 ​μM (∗∗∗p ​= ​0.0001). Similarly, TNFα transcript levels were significantly reduced at both 1 ​μM and 10 ​μM (∗∗∗∗p ​< ​0.0001).

A hallmark of innate immunity in *Drosophila* is the production and release into the hemolymph of antimicrobial peptides (AMPs) exerting inflammatory response regulation and antimicrobial actions [35,36]. This process, sustaining a systemic response after cellular stress or pathogen contact, is orchestrated by the immunodeficiency pathway, which is regulated by NF-κB-like factors. Given our previous demonstration of up-regulated AMPs in flies with pan-neuronal expression of the ALS-associated A4V and G85R *hSOD1* mutations [22], we investigated the effect of 0.5 ​mM clomipramine on AMPs transcript expression. We quantified the expression levels of the AMPs attacin, cecropin, diptericin, and drosocin, which were the four most highly up-regulated among those analyzed in both mutant models, in heads of untreated and treated ALS flies. As shown in Fig. 3A, clomipramine significantly lowered all tested AMPs in both *hSOD1*^*A4V*^ and *hSOD1*^*G85R*^ models.

Assumed the shared roles of *Drosophila* AMPs and mammalian cytokines in innate immunity and inflammation, we next investigated whether clomipramine treatment could similarly modulate the expression of the pro-inflammatory interleukin-1β (IL-1β) and tumor necrosis factor α (TNFα). To this aim, we leveraged the murine motor neuron-like *hSOD1*^*G93A*^-NSC-34 ​cell line [37], the predominant *in vitro* model to investigate ALS pathogenesis [38]. As shown in Fig. 3B, clomipramine significantly reduced the expression of both pro-inflammatory cytokines at 10 ​μM, with effects also observed at a concentration as low as 1 ​μM.

These results indicate that the anti-inflammatory action of clomipramine extends to the A4V, G85R, and G93A *SOD1* mutations.

### Clomipramine attenuates DNA damage in SOD1-ALS flies

**Fig. 4:**
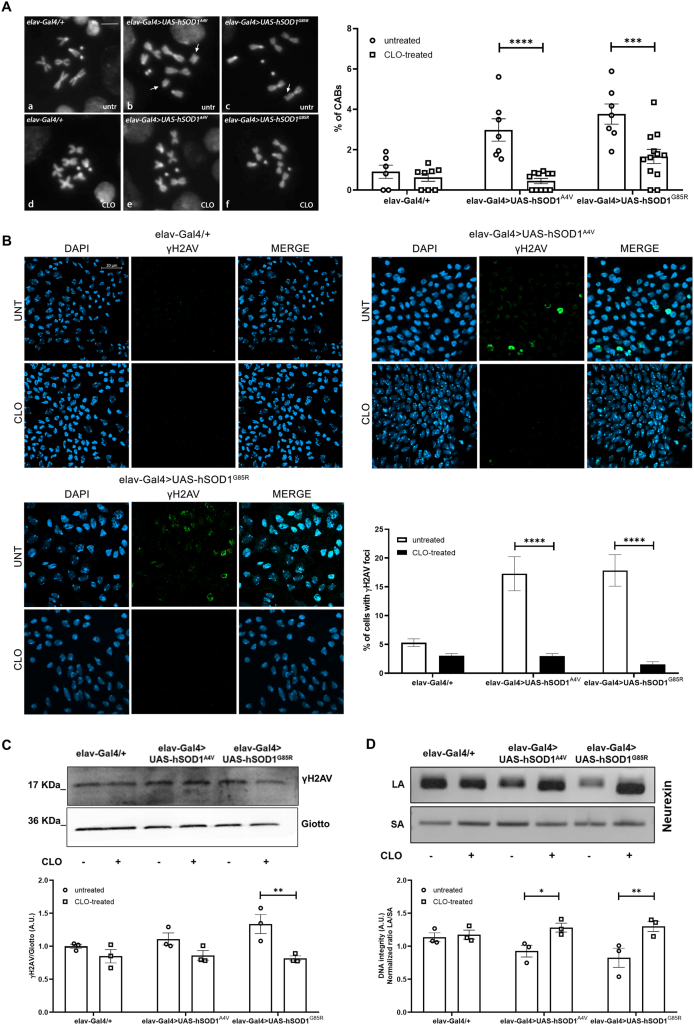
**Clomipramine attenuates DNA damage in *hSOD1*-ALS flies**. **A)** Representative images of metaphase chromosomes from non-mutant and ALS third instar *Drosophila* larval brains, untreated (a, b, c) and 0.5 ​mM clomipramine-treated (d, e, f). Panels a, d, e, and f show normal karyotypes; panel b shows an isochromatid centromere break (arrows); panel c reports a chromatid deletion (arrow). Scale bar 5 ​μm. On the right side, bar graphs indicate the number (%) of chromosome aberrations (CABs) ​± ​SEM. Individual untreated (circles) and clomipramine-treated (squares) data points represent each analyzed larval brain (at least 500 ​cells were scored for each brain). Statistical significance was determined using a two-way ANOVA to evaluate the effects of genotype (F_(2, 45)_ ​= ​14.27, p ​< ​0.0001), treatment (F_(1, 45)_ ​= ​30.99, p ​< ​0.0001) and their interaction (F_(2, 45)_ ​= ​5.059, p ​= ​0.0104). Sidak's multiple comparisons test confirms that clomipramine significantly reduced CABs in both *hSOD1*^*A4V*^ (∗∗∗∗p ​< ​0.0001) and *hSOD1*^*G85R*^ (∗∗∗p ​< ​0.001) larvae. **B)** Confocal microscopy images of γH2AV foci (green) in untreated and 0.5 ​mM clomipramine-treated non-mutant (*elav-Gal4/+*) and ALS (*elav-Gal4>UAS-hSOD1*^*A4V*^ and *elav-Gal4>UAS-hSOD1*^*G85R*^) larval brains. DAPI was used to stain nuclei. Scale bar 20 ​μm. Bar graphs indicate the number (%) ​± ​SEM of γH2AV-positive cells. Statistical significance was calculated by two-way ANOVA test with genotype (F_(2,54)_ ​= ​7.682, p ​= ​0.0012) and clomipramine treatment as main factors (F_(1,54)_ ​= ​62.62, p ​< ​0.0001). A significant genotype ​× ​treatment interaction (F_(2,54)_ ​= ​10.00, p ​= ​0.0002) was observed. Sidak's multiple comparisons test confirms that clomipramine significantly reduced the number of γH2AV-positive cells in both *hSOD1*^*A4V*^ and *hSOD1*^*G85R*^ (∗∗∗∗p ​< ​0.0001) larvae. Scored cells: *elav-Gal4/+* ​= ​2385; *elav-Gal4/+* CLO ​= ​2415; *elav-Gal4>UAS-hSOD1*^*A4V*^ ​= ​2762; *elav-Gal4>UAS-hSOD1*^*A4V*^ CLO ​= ​3968; *elav-Gal4>UAS-hSOD1*^*G85R*^ ​= ​3092; *elav-Gal4>UAS-hSOD1*^*G85R*^ CLO ​= ​2010. **C)** Western blot analysis of *Drosophila* γH2AV expression in untreated (circles) and clomipramine-treated (squares) non-mutant (*elav-Gal4/+*) and ALS (*elav-Gal4>UAS-hSOD1*^*A4V*^ and *elav-Gal4>UAS-hSOD1*^*G85R*^) adult fly heads. Giotto protein was used as loading control. Bar graphs represent the mean ​± ​SEM of the normalized ratio between γH2AV and Giotto obtained by three independent biological replicates, as represented by individual data points. Two-way ANOVA showed a significant main effect of treatment (F_(1, 12)_ ​= ​17.79, ∗∗p ​= ​0.0012), but no significant main effect of genotype (F_(2, 12)_ ​= ​1.495, p ​= ​0.2632) or interaction (F_(2, 12)_ ​= ​2.331, p ​= ​0.1395) was observed. Sidak's multiple comparisons test reported a statistically significant decrease in γH2AV expression specifically in the clomipramine-treated *hSOD1*^*G85R*^ group compared to its untreated control (∗∗p ​< ​0.01). **D)** Agarose gel image of long (∼8.8 ​kb, LA) and short (∼200 bp, SA) amplicons of the *Neurexin* gene obtained amplifying genomic DNA isolated from non-mutant (*elav-Gal4/+*) and ALS (*elav-Gal4>UAS-hSOD1*^*A4V*^ and *elav-Gal4>UAS-hSOD1*^*G85R*^) untreated (circles) and clomipramine-treated (squares) adult fly heads. Bar graphs on the right show the mean ​± ​SEM of relative DNA integrity resulting by LA/SA ratio obtained by three independent biological replicates, as indicated by individual data points. Two-way ANOVA revealed a significant main effect of the clomipramine (F_(1, 12)_ ​= ​15.66, p ​= ​0.0019), while no significant main effect of genotype (F_(2, 12)_ ​= ​0.5204, p ​= ​0.6071) or interaction (F_(2, 12)_ ​= ​3.135, p ​= ​0.0803) was observed. Sidak's multiple comparisons test showed that clomipramine treatment significantly increased the LA/SA ratio in both the *hSOD1*^*A4V*^ (∗p ​< ​0.05) and *hSOD1*^*G85R*^ (∗∗p ​< ​0.01) ALS fly models compared to their respective untreated controls.

We previously demonstrated that pan-neuronal expression of *hSOD1*^*A4V*^ or *hSOD1*^*G85R*^ mutations induces chromosome aberrations (CABs) in third-instar-larval neuroblasts (the only actively replicating neuronal cells from which metaphase chromosomes can be isolated) [22]. To test if clomipramine could rescue chromosome damage, we analyzed DAPI-stained metaphase chromosomes obtained from 0.5 ​mM clomipramine-fed *hSOD1*^*A4V*^ and *hSOD1*^*G85R*^ larvae. As reported in Fig. 4A, treatment with clomipramine favorably and significantly affected CABs frequency in both *hSOD1*^*A4V*^ (84 ​% reduction - A4V ​= ​2.97 ​% vs A4V CLO ​= ​0.45 ​%) and *hSOD1*^*G85R*^ (56 ​% reduction – G85R ​= ​3.76 ​% vs G85R CLO ​= ​1.66 ​%) larvae.

To further corroborate these findings and investigate the underlying clomipramine-sensitive mechanisms of genomic instability, we measured the number of phosphorylated histone H2AV (γH2AV) foci, i.e. microscopically visible clusters of γH2AV protein. These are well-established markers for DNA double-strand breaks and key indicators of primary DNA damage [39,40], whose increase directly reflects the presence of DNA lesions that, if unrepaired, can lead to CABs. Confocal images reported in Fig. 4B confirm clomipramine efficacy in mitigating the formation of γH2AV foci, determining respectively 82 ​% decrease (A4V ​= ​17.27 ​% vs A4V CLO ​= ​2.97 ​%) in *hSOD1*^*A4V*^ and 91 ​% (G85R ​= ​17.84 ​% vs G85R CLO ​= ​1.51 ​%) in *hSOD1*^*G85R*^ larvae.

In order to comprehensively extend the potential effect of clomipramine on genome integrity, we investigated the presence of DNA damage in adult flies. We performed Western blot analysis of γH2AV protein on heads isolated from untreated and 0.5 ​mM clomipramine-treated flies. As reported in Fig. 4C, clomipramine significantly reduced γH2AV levels by 38 ​% in *hSOD1*^*G85R*^ flies. While a tendency for a decrease was also observed in *hSOD1*^*A4V*^ flies, this was not statistically significant. This result suggests a *hSOD1*^*G85R*^ mutation-specific protective role of clomipramine against cellular stress.

Beyond immediate DNA lesions, we also examined the transcription-associated consequences of DNA damage by performing long-amplicon (LA) PCR on genomic DNA obtained from adult heads of untreated and treated *hSOD1* flies. It is well-known that large deletions impede polymerase progression and result in reduced amplification efficiency of long DNA fragments [41]. We executed LA-PCR on the extensive amplicon (>8.8 ​kB) of *Neurexin* gene, whose large size makes it particularly sensitive to potential DNA damage. Agarose gel in Fig. 4D shows that clomipramine significantly ameliorated amplification efficiency in both *hSOD1*^*A4V*^ and *hSOD1*^*G85R*^ adult heads. The lack of a significant effect of genotype, as shown by two-way ANOVA, suggests that while the *hSOD1* mutations do not cause a measurable reduction in DNA integrity, they create a genomic vulnerability that is mitigated by clomipramine.

Taken together, these results strongly suggest the protective role that clomipramine exerts against genomic instability, by reducing both primary and secondary DNA lesions.

### Drug repurposing validation of additional drug candidates

Our drug repurposing investigation identified not only clomipramine, but also mianserin (Lantanon®/Tolvon® - 1, 2, 3, 4, 10, 14b-Hexahydro-2-methyldibenzo[c, f]pyrazino[1, 2-a] azepine) and modafinil (Provigil® - (RS)-2-(Diphenylmethylsulfinyl)acetamide) as potential therapeutic candidates for ALS treatment [15]. Mianserin is a tetracyclic compound currently used for the treatment of depression and anxiety [42] (Table 2). The antidepressant activity is due to its action as a serotonin receptor antagonist (5-HT2A, 5-HT2C, 5-HT3), combined with a sedative effect, supported by its antagonism at the H1 histamine receptor [43].

**Table 2:** **Pharmacological profile of mianserin.** The table summarizes some key pharmacological characteristics of mianserin (Drug Bank ID: DB06148), including its chemical structure, established clinical indications and primary mechanisms of action.

Structure	Indications	Mechanisms of action
	- Depression - Anxiety	**Reuptake inhibitor:** - Serotonin - Dopamine - Noradrenaline (weak) **Antagonist:** - 5-HT1A, 5-HT1F, 5-HT2A, 5-HT2B, 5-HT2C, 5-HT6, 5-HT7 serotonin receptors - H1 and H4 histamine receptors - α1, α2A, α2B, α2C adrenergic receptors - D1, D2, D3 dopamine receptors

**Fig. 5:**
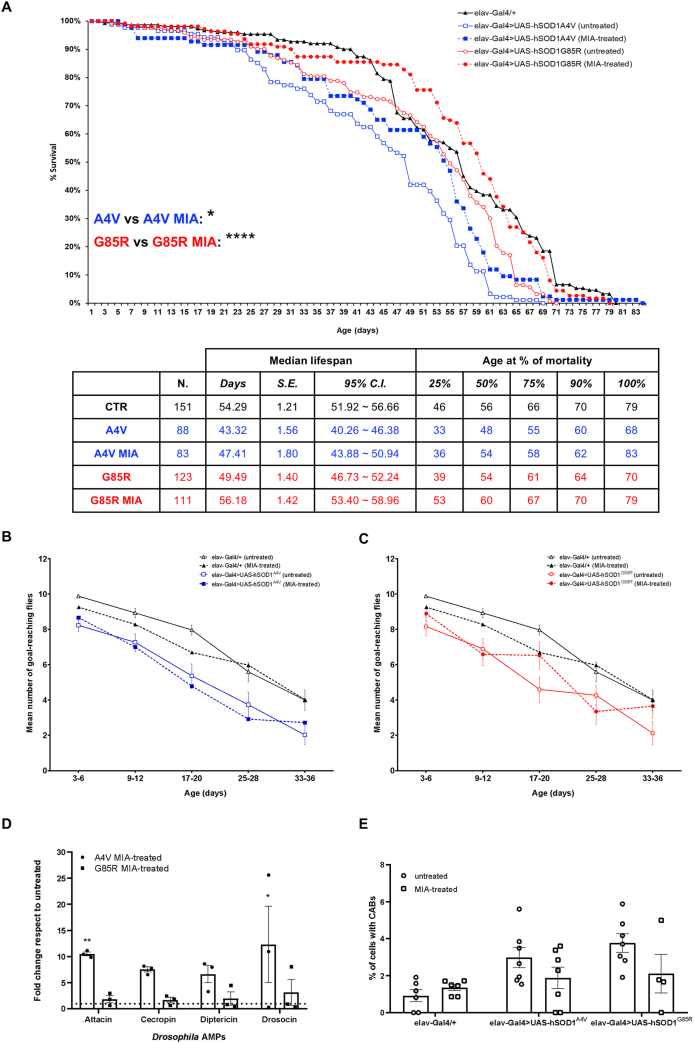
**Mianserin ameliorates survival but exacerbates inflammation in *hSOD1*^*A4V*^ and *hSOD1*^*G85R*^ ALS flies**. **A)** Survival curves of: *elav-Gal4/+* (CTR, black line, black triangles, n ​= ​151), *elav-Gal4>UAS-hSOD1*^*A4V*^ untreated (A4V, blue line, empty blue squares, n ​= ​88) and mianserin-treated (A4V MIA, blue line, full blue squares, n 83), *elav-Gal4>hSOD1*^*G85R*^*untreated* (G85R, red line, empty red circles, n ​= ​123) and mianserin-treated (G85R MIA, red line, full red circles, n ​= ​111). Data are pooled from three independent biological replicates, each originating from separate parental crosses. Statistical significance was calculated by Log-rank test with Bonferroni correction through OASIS2 online software, A4V vs A4V MIA: ∗p ​< ​0.05, G85R vs G85R MIA: ∗∗∗∗p ​< ​0.0001. The table summarizes the number of flies analyzed in mianserin-treated survival curves and their respective controls, median lifespan ​± ​S.E. (standard error) with 95 ​% C.I. (confidence interval), and fly age (in days) at 25 ​%, 50 ​%, 75 ​%, 90 ​%, and 100 ​% mortality for each genotype. **B)** Negative geotaxis assay at different time-points (3–6, 9–12, 17–20, 25–28 and 33–36 days post-eclosion) of untreated (black line, empty black triangles) and mianserin-treated (black line, full black triangles) *elav-Gal4/+* flies and untreated (blue line, empty blue squares) and treated (blue line, full blue squares) *elav-Gal4>UAS-hSOD1*^*A4V*^ flies. Statistical significance was calculated by three-way mixed-design ANOVA (time as repeated factor) followed by Tukey's *post hoc* test for multiple comparisons. The overall ANOVA revealed highly significant main effects for time (F_(3.14, 113.3)_ ​= ​119.6, p ​< ​0.0001) and genotype (F_(1,36)_ ​= ​22.67, p ​< ​0.0001). However, no significant main effect was found for treatment (p ​= ​0.4970), genotype ​× ​treatment interaction (p ​= ​0.6719), or the three-way interaction (p ​= ​0.3982). Tukey's *post hoc* test did not detect significance in the direct comparisons between treated and untreated groups at corresponding time-points. **C)** Negative geotaxis assay at different time-points (3–6, 9–12, 17–20, 25–28 and 33–36 days post-eclosion) of untreated (black line, empty black triangles) and mianserin-treated (black line, full black triangles) *elav-Gal4/+* flies and untreated (red line, empty red circles) and treated (red line, full red circles) *elav-Gal4>UAS-hSOD1*^*G85R*^ flies. Statistical significance was calculated by three-way mixed-design ANOVA (time as repeated factor) followed by Tukey's *post hoc* test for multiple comparisons. The overall ANOVA revealed highly significant main effects for time (F_(3.58, 128.9)_ ​= ​122.8, p ​< ​0.0001), genotype (F_(1,36)_ ​= ​15.02, p ​= ​0.0004) and genotype x treatment ​× ​time interaction (F_(4, 144)_ ​= ​4.19, p ​= ​0.0031). No significant main effect was found for treatment (p ​= ​0.8218) and genotype ​× ​treatment interaction (p ​= ​0.2123). Tukey's *post hoc* test did not detect significance in the direct comparisons between treated and untreated groups at corresponding time-points. In **B)** and **C)** at least 100 flies (pooled from three separate parental crosses) for each genotype/treatment were analyzed. Locomotor ability is represented as the mean number of flies reaching the goal of 8 ​cm from the bottom of the vial within 10 ​s ​± ​SEM. Each vial was tested three times. **D)** qPCR analysis of AMPs transcripts (attacin, cecropin, diptericin and drosocin) in *elav-Gal4>UAS-hSOD1*^*A4V*^ (circles) and *elav-Gal4>hSOD1*^*G85R*^ (squares) mianserin-treated adult fly heads. Transcript levels were normalized to *actin* gene and are represented as fold change relative to untreated genotype-matched flies (dotted horizontal line set to 1). Bar graphs represent the mean ​± ​SEM of three independent biological replicates each with three technical replicates, as indicated by individual data points. Statistical significance was calculated by two-way ANOVA and revealed a significant main effect of genotype (F_(2,24)_ ​= ​14.64, p ​< ​0.0001) and no main effect of the AMP gene (F_(3, 24)_ ​= ​0.6088, p ​= ​0.6158) nor interaction AMP gene x treatment (F_(6, 24)_ ​= ​0.3698, p ​= ​0.8909). Dunnett's multiple comparisons test reported a significant over-expression of attacin (∗∗p ​= ​0.0042) and drosocin (∗p ​= ​0.0156) in *elav-Gal4>UAS-hSOD1*^*A4V*^ flies. **E)** Bar graph reports the percentage ​± ​SEM of chromosome abnormalities scored in metaphase chromosomes obtained from third instar non-mutant (*elav-Gal4/+*) and ALS (*elav-Gal4>UAS-hSOD1*^*A4V*^ and *elav-Gal4>UAS-hSOD1*^*G85R*^) larval brains. Individual data points of untreated (circles) and mianserin-treated (squares) represent each analyzed brain (for each brain at least 500 ​cells were scored). Statistical significance was obtained by two-way ANOVA that revealed, as expected, a statistically significant effect of the genotype (F_(2,31)_ ​= ​5.634, p ​= ​0.0082), while neither effect of the treatment (F_(1,31)_ ​= ​3.019, p ​= ​0.0922) nor the interaction genotype x treatment (F_(2,31)_ ​= ​1.926, p ​= ​0.1628) were reported. The *post hoc* Sidak's multiple comparisons test showed no significant difference in chromosome abnormalities between treated and untreated samples (p ​= ​0.1567).

**Fig. S3:**
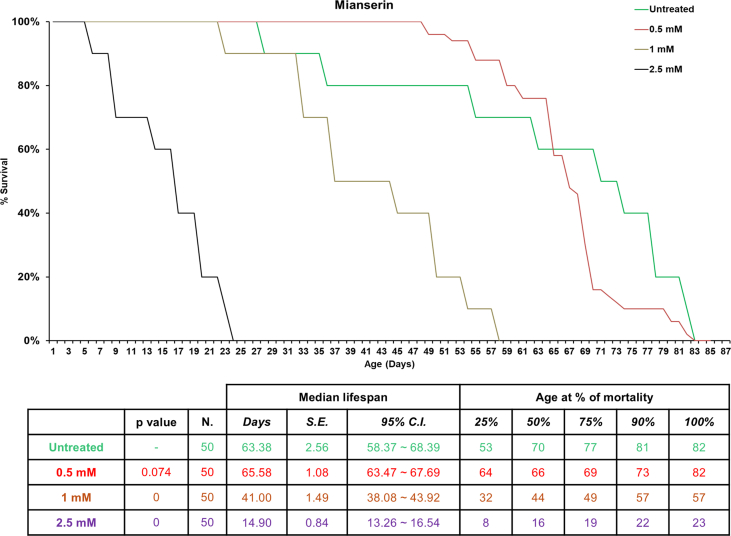
**Survival curves of wild type flies to determine the optimal dose of mianserin.** Survival curves of 50 wild type *OregonR* flies administered with mounting doses of mianserin (0.5 ​mM – red line; 1 ​mM – brown line; 2.5 ​mM black line). Green line indicates the survival of the flies reared on normal medium. 0.5 ​mM mianserin was selected because it caused no toxicity compared to vehicle (Bonferroni p value ​= ​0.074). All higher concentrations induced acute toxicity (p ≈ 0 for all comparisons). The table summarizes the number of flies analyzed, the Bonferroni p value, their median lifespan ​± ​S.E. (standard error) with 95 ​% C.I. (confidence interval), and fly age (in days) at 25 ​%, 50 ​%, 75 ​%, 90 ​%, and 100 ​% mortality for each tested concentration.

Once established the optimal dose of mianserin based on tolerability studies (0.5 ​mM, Supp. Fig. 3), we tested its effects in our SOD1-*Drosophila* models. Mianserin exhibited the most beneficial effect on fly survival compared to clomipramine and modafinil (Table 4 and described below). It extended the lifespan of *hSOD1*^*A4V*^ flies by 22 ​% and of *hSOD1*^*G85R*^ by 13 ​% (Fig. 5A). Regarding locomotor function, mianserin's impact was complex. While it had no significant effect on *hSOD1*^*A4V*^ locomotor activity (Fig. 5B), three-way ANOVA of the *hSOD1*^*G85R*^ climbing performance revealed a highly significant time ​× ​genotype ​× ​treatment interaction (p ​= ​0.0031), indicating that the effect of mianserin on motility is highly dependent on both the time-point and the underlying genotype (Fig. 5C). However, *post hoc* analysis did not detect significant differences in direct comparisons between treated and untreated flies at specific time-points. Furthermore, we observed that mianserin did not suppress the overall inflammation in either *hSOD1* model and, indeed, in *hSOD1*^*A4V*^ flies, it determined a significant up-regulation of attacin and drosocin AMPs (Fig. 5D). Moreover, we also observed a non-significant trend toward reducing chromosome breaks (Fig. 5E). The observed variability and lack of consistent efficacy across multiple fly phenotypes is supported by literature, where mianserin was already noted to require further validation in the context of survival [44].

**Table 3:** **Pharmacological profile of modafinil.** The table summarizes some key pharmacological characteristics of modafinil (Drug Bank ID: DB00745), including its chemical structure, established clinical indications, off-label uses and primary mechanisms of action.

Structure	Indications	Off-label uses	Mechanisms of action
	- Narcolepsy - Sleep apnea - Shift worker disorder	- ADHD - Depression - Fatigue	**Reuptake inhibitor:** - Dopamine **Antagonist:** - α1 adrenergic receptors

**Table 4:**
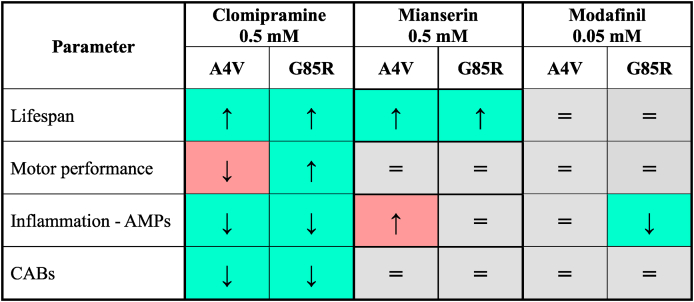
**Summary view.** The table schematizes the effects of clomipramine, mianserin and modafinil on selected biological parameters in *hSOD1*^*A4V*^ ​or *hSOD1*^*G85R*^ ​ALS flies. Upregulation is represented by ↑ arrow, downregulation by ↓ arrow, unchanged or not significant results by =. Green background stands for beneficial effect, red for detrimental, and gray for neutral/unchanged.

Modafinil is a bicyclic compound used for the treatment of narcolepsy [45] (Table 3) and increasing the release of histamine from the hypothalamus, by attenuating inhibitory GABAergic input to histamine neurons [46,47]. Furthermore, modafinil inhibits dopamine reuptake, inactivates GABAergic and activates glutamatergic circuits [48,49].

**Fig. 6:**
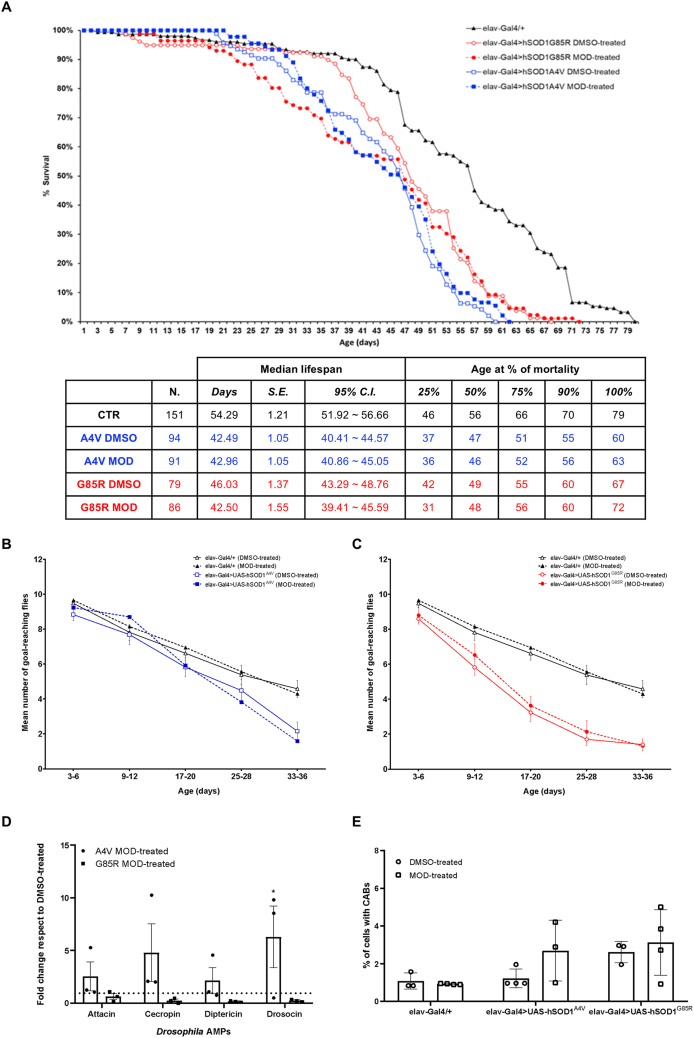
**Modafinil does not ameliorate ALS phenotypes in *hSOD1*^*A4V*^ and *hSOD1*^*G85R*^ flies. A)** Survival curves of: *elav-Gal4/+* (CTR, black line, black triangles, n ​= ​151), *elav-Gal4>UAS-hSOD1*^*A4V*^ DMSO-treated (A4V, blue line, empty blue squares, n ​= ​94) and modafinil-treated (A4V MOD, blue line, full blue squares, n ​= ​91), *elav-Gal4>hSOD1*^*G85R*^ DMSO-treated (G85R, red line, empty red circles, n ​= ​79) and modafinil-treated (G85R MOD, red line, full red circles, n ​= ​86). Data are pooled from three independent biological replicates, each originating from separate parental crosses. Statistical significance was calculated by Log-rank test with Bonferroni correction through OASIS2 online software. The table summarizes the number of flies analyzed in modafinil-treated survival curves and their respective controls in DMSO, median lifespan ​± ​S.E. (standard error) with 95 ​% C.I. (confidence interval), and fly age (in days) at 25 ​%, 50 ​%, 75 ​%, 90 ​%, and 100 ​% mortality for each genotype. **B)** Negative geotaxis assay at different time-points (3–6, 9–12, 17–20, 25–28 and 33–36 days post-eclosion) of DMSO-treated (black line, empty black triangles) and modafinil-treated (black line, full black triangles) *elav-Gal4/+* flies and DMSO-treated (blue line, empty blue squares) and treated (blue line, full blue squares) *elav-Gal4>UAS-hSOD1*^*A4V*^ flies. Statistical significance was calculated by three-way mixed-design ANOVA (time as repeated factor). The overall ANOVA revealed highly significant main effects for time (F_(2.917, 105.0)_ ​= ​123.0, p ​< ​0.0001) and genotype (F_(1,36)_ ​= ​15.67, p ​= ​0.0003), confirming the age-dependent decline and the difference between the mutant and control lines. Furthermore, a highly significant time ​× ​genotype interaction was observed (F_(4,144)_ ​= ​5.427, p ​= ​0.0004), indicating that the rate of motor decline differs significantly between the two genotypes. No significant main effect was found for treatment (p ​= ​0.6796), nor were the genotype ​× ​treatment (p ​= ​0.8632) or the three-way interactions significant (p ​= ​0.7950). Tukey's *post hoc* test did not detect significance in the direct comparisons between treated and untreated groups at corresponding time-points. **C)** Negative geotaxis assay at different time-points (3–6, 9–12, 17–20, 25–28 and 33–36 days post-eclosion) of DMSO-treated (black line, empty black triangles) and modafinil-treated (black line, full black triangles) *elav-Gal4/+* flies and DMSO-treated (red line, empty red circles) and treated (red line, full red circles) *elav-Gal4>UAS-hSOD1*^*G85R*^ flies. Statistical significance was calculated by three-way mixed-design ANOVA (time as repeated factor). The overall ANOVA revealed highly significant main effects for time (F_(3.643, 138.4)_ ​= ​197.5, p ​< ​0.0001) and genotype (F_(1,38)_ ​= ​66.83, p ​< ​0.0001), confirming the age-dependent decline and the difference between the mutant and control lines. Furthermore, a highly significant time ​× ​genotype interaction was observed (F_(4,152)_ ​= ​10.21, p ​< ​0.0001), indicating that the rate of motor decline differs significantly between the two genotypes. No significant main effect was found for treatment (p ​= ​0.4295), nor were the genotype ​× ​treatment (p ​= ​0.7625) or the three-way interactions significant (p ​= ​0.9972). Tukey's *post hoc* test did not detect significance in the direct comparisons between treated and untreated groups at corresponding time-points. In **B)** and **C)**, at least 100 flies (pooled from three separate parental crosses) for each genotype/treatment were analyzed. Locomotor ability is represented as the mean number of flies reaching the goal of 8 ​cm from the bottom of the vial within 10 ​s ​± ​SEM. Each vial was tested three times. **D)** qPCR analysis of AMPs transcripts (attacin, cecropin, diptericin and drosocin) in *elav-Gal4>UAS-hSOD1*^*A4V*^ (circles) and *elav-Gal4>hSOD1*^*G85R*^ (squares) modafinil-treated adult fly heads. Transcript levels were normalized to *actin* gene and are represented as fold change relative to DMSO-treated genotype-matched flies (dotted horizontal line set to 1). Bar graphs represent the mean ​± ​SEM of three independent biological replicates, as indicated by individual data points, each with three technical replicates. Statistical significance was calculated by two-way ANOVA and revealed a significant main effect of genotype (F_(2,24)_ ​= ​9.143, p ​= ​0.0011) and no main effect of the AMP gene (F_(3, 24)_ ​= ​0.7343, p ​= ​0.5418) nor interaction AMP gene x treatment (F_(6, 24)_ ​= ​0.8213, p ​= ​0.5645). Dunnett's multiple comparisons test reported a significant over-expression of drosocin (∗p ​= ​0.0135) in *elav-Gal4>UAS-hSOD1*^*A4V*^ flies. **E)** Bar graph reports the percentage ​± ​SEM of chromosome abnormalities scored in metaphase chromosomes obtained from third instar non-mutant (*elav-Gal4/+*) and ALS (*elav-Gal4>UAS-hSOD1*^*A4V*^ and *elav-Gal4>UAS-hSOD1*^*G85R*^) larval brains. Individual data points from DMSO-treated (circles) and modafinil-treated (squares) brains represent each analyzed brain (for each brain at least 500 ​cells were scored). Statistical significance was calculated by two-way ANOVA test that revealed, as expected, a statistically significant effect of the genotype (F_(2,15)_ ​= ​5.601, p ​= ​0.0152), while neither effect of the treatment (F_(1,15)_ ​= ​1.783, p ​= ​0.2017) nor the interaction genotype x treatment (F_(2,15)_ ​= ​1.092, p ​= ​0.3609) were reported. The *post hoc* Sidak's multiple comparisons test showed no significant difference in chromosome abnormalities between treated and untreated samples.

**Fig. S4:**
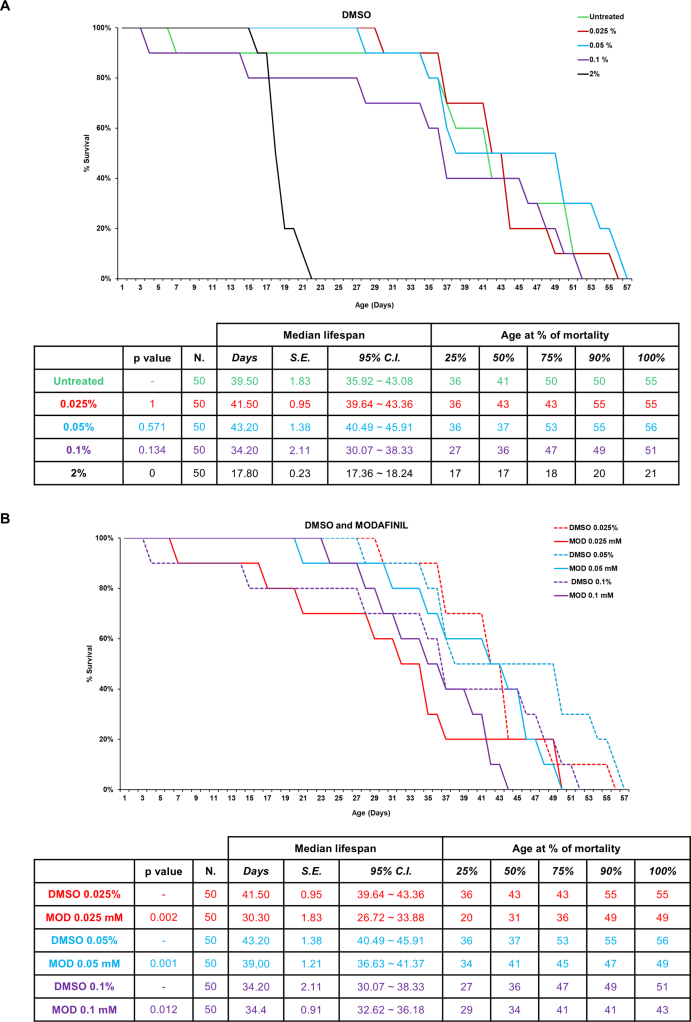
**Survival curves of wild type flies to determine the optimal dose of DMSO and modafinil. A)** Survival curves of 50 wild type *OregonR* flies administered with mounting doses of DMSO (0.025 ​% – red line; 0.05 ​% - blue line; 0.1 ​% – purple line; 2 ​% – black line). Green line indicates the survival of the flies reared on normal medium. All DMSO concentrations showed no statistical toxicity compared to vehicle (0.025 ​% p ​= ​1; 0.05 ​% p ​= ​0.571; 0.1 ​% p ​= ​0.134), exception made for 2 ​% DMSO (p ≈ 0). **B)** Survival curves of 50 wild type *OregonR* flies administered with mounting doses of modafinil (mM, full line) or DMSO (%, dotted line) (0.025– red line; 0.05 - blue line; 0.1– purple line). All three tested modafinil concentrations induced a slight, statistically significant toxicity (0.025 ​mM p ​= ​0.002; 0.05 ​mM p ​= ​0.001; 0.1 ​mM p ​= ​0.012) compared to their correspondent DMSO control. The 0.05 ​mM dose was chosen for subsequent experiments as it maintained toxicity below the pre-defined threshold of less than 15 ​% loss of median lifespan relative to DMSO control. In **A)** and in **B)** tables summarizes the number of flies analyzed, the Bonferroni p value, their median lifespan ​± ​S.E. (standard error) with 95 ​% C.I. (confidence interval), and fly age (in days) at 25 ​%, 50 ​%, 75 ​%, 90 ​%, and 100 ​% mortality for each tested concentration.

Despite being modafinil the drug with the best score emerged from the SAveRUNNER analysis [15], the treatment of *hSOD1* flies with its optimal dose based on tolerability assays (0.05 ​mM, Supp. Fig. 4) did not demonstrate beneficial effects on lifespan extension, locomotor activity and genomic instability (Fig. 6A–C, E). Its unique effects were a favorable trend reduction in AMP transcripts only in *hSOD1*^*G85R*^ flies and a significant detrimental increase in drosocin AMPs (Fig. 6D).

## Discussion

Compelling evidence now regards ALS as an active and dynamic disease, characterized by a multipart interplay of neurodegenerative, inflammatory, metabolic, and DNA damage actions that gradually reshape and compromise homeostasis until an irreversible breaking point [50]. Given the multigenic, multisystemic, non-cell-autonomous, multifactorial nature of ALS [1,51,52], and the regrettable failure of all therapies in use so far [53,54], there is imperative necessity of introducing a drastic shift in how the disease is managed regarding targeted disease-modifying interventions and therapeutic strategies. While frustrating on one side, the lack of effective drugs stimulates further scientific research. Indeed, in recent years, several studies have attempted *in silico* and *in vivo* approaches for repurposing various compounds against ALS. Specifically, a study utilizing genetic analysis and computational prioritization provided crucial support for compounds targeting well-known pathways (like RAS/RAF/MAPK and vitamin B signaling) and suggested that for instance BRAF inhibitors might be beneficial, either alone or in combination with trametinib [55]. A purely *in silico* investigation focusing on the *SOD1*^*C146R*^ mutation, identified two FDA-approved drugs, regorafenib and phenylpropanolamine, based on their strong binding affinity to the dimeric SOD1 structure [56]. Finally, an *in vivo* study provided evidence that the anti-tumoral 5-fluorouracil significantly increases lifespan and delays disease onset in the *hSOD1*^*G93A*^ mouse model of ALS [57]. Across all these approaches, the ultimate consensus remains that all candidates require further extensive preclinical and clinical validation. Of note, several compounds with distinct original indications are currently in Phase II/III trials for repurposing in ALS treatment [58]. In this perspective, our network medicine and drug-repurposing approach has recently identified clomipramine, mianserin, and modafinil to be preclinically tested against ALS, among hundreds of different compounds that emerged as potentially repurposable [15]. This was the aim of the present work, the preclinical validation of *in silico-*emerged drugs.

Within the limitation of the two *Drosophila* models used here (for instance no correlation between mutant *hSOD1* temporal expression in fly and ALS disease insurgence and progression in patients), characterized by pan-neuronal expression of *hSOD1*^*A4V*^ and *hSOD1*^*G85R*^ mutations [22], our report provides new evidence about the potential role of clomipramine against ALS. Our characterization revealed that clomipramine presents the most complex and intriguing profile among the tested compounds, likely due to its multi-target nature (Table 4).

Indeed, the tricyclic antidepressant clomipramine (registered on the World Health Organization's list as an essential medicine, https://iris.who.int/handle/10665/325771) is a potent inhibitor of serotonin transporter (SERT) and norepinephrine transporter causing their increased concentration in the synaptic cleft [17,18]. Moreover, it elicits anti-adrenergic (α1-R), anti-histaminergic (H1-R), anti-serotoninergic (5-HT2A, 2B, 2C, 3, 6, 7-R), anti-dopaminergic (D1, 2, 3-R), and anti-cholinergic (M1, 2, 3-R) activities [59,60]. As other tricyclic antidepressants, clomipramine slightly blocks also voltage-dependent sodium channels [61], thus boosting its nature as a multi-target directed ligand. With no doubt this feature sustains its potential power against ALS, a disease requiring a multi-drug approach being characterized not only by motor neuron degeneration in motor cortex/spinal cord, but also by clinical dysregulations of sensory neurons [62], sympathoadrenal axis [63], mesencephalic trigeminal neurons [64], with impaired functioning of glutamate, GABA, and the previously mentioned neurotransmitter circuits that are sensitive to clomipramine modulation.

According to the preclinical analysis shown here, the susceptibility to clomipramine has highlighted that the progression of symptoms from larval to adult state culminating into motor impairment and reduced lifespan (both improved by clomipramine in *hSOD1*^*A4V*^ and *hSOD1*^*G85R*^ *Drosophila*), is not a matter of time-dependent deterioration, but it is instead associated with multiple systemic, cellular, and molecular events including DNA damage and inflammatory imbalance (remarkably ameliorated by clomipramine treatment). These alterations, if not mitigated by clomipramine, overall participate in a pro-inflammatory microenvironment, thus amplifying pathological remodeling and accelerating disease evolution.

A peculiar result emerged from our analysis is the stronger effect exerted by clomipramine on *hSOD1*^*A4V*^ respect to *hSOD1*^*G85R*^ fly survival, accompanied by an opposite modulation of motor performance, respectively detrimental or beneficial, in *hSOD1*^*A4V*^ or *hSOD1*^*G85R*^ flies. We reported that the worsened motor performance by clomipramine correlated with a significant reduction in AChE activity in *hSOD1*^*A4V*^ flies. Chronic administration of several antidepressants may indeed inhibit AChE and lead to an acetylcholine increase at the neuromuscular synapse, in turn inducing the so-called “cholinergic effect” [65,66]. While an initial increase in acetylcholine can enhance muscle stimulation, prolonged overstimulation can paradoxically reduce muscle strength and contraction, resulting in muscle weakness or paralysis [67]. Interestingly, the role of AChE deficiency has been previously investigated in a zebrafish ALS model and in plasma of patients, highlighting the importance of AChE alterations during the early stages of ALS and leading to a reconsideration of its contribution to pathological outcomes [68,69]. On the other hand, the divergent responses to clomipramine between the two SOD1 fly models might emphasize an aspect of ALS that is becoming increasingly evident. Even though mutated *SOD1* gene determines the core of ALS pathogenesis, each specific mutation is responsible for distinct phenotypic nuances (in our case different sensitivity to clomipramine). The two mutants studied here represent distinct pathological classes: the *A4V* substitution is clinically the most aggressive *SOD1* variant, characterized by extreme structural instability and aggregation, leading to rapid motor neuron loss. Conversely, the *G85R* mutant is primarily associated with defects in metal binding and a more pronounced non-cell-autonomous toxicity [[70], [71], [72]]. These differences may likely contribute to the divergent pharmacological responses to clomipramine that will need to be fully and carefully elucidated to ensure more efficacious and tailored therapy [[73], [74], [75]].

Another key result emerged from our present work is the anti-inflammatory action of clomipramine. Inflammation, and in particular the activation of the innate immune response, is a well-established pathological hallmark of ALS [[76], [77], [78]]. The convergence of data from *in vivo* and *in vitro* models showing significant reduction in the expression of AMPs in *hSOD1-*flies and pro-inflammatory cytokines (IL-1β and TNFα) in *hSOD1*^*G93A*^-transfected NSC-34 motor neuron cells confirms a protective role for clomipramine in modulating inflammation. This is consistent with previous studies highlighting anti-inflammatory effects of clomipramine in additional models of neurological and inflammatory diseases, such as multiple sclerosis [19], ulcerative colitis [20], and also during the SARS-CoV2 infection-dependent cytokine storm [79].

Particularly relevant in our *in vivo* analysis is also the demonstration that clomipramine attenuates DNA damage and genomic instability induced by both *SOD1* mutations. Maintaining genomic integrity is crucial for neuronal survival, and the accumulation of DNA damage is increasingly recognized as an important pathogenic feature of ALS [[80], [81], [82], [83]]. CABs and γH2AV foci reduction by clomipramine in larval neuroblasts, combined with lowered rate of γH2AV and higher DNA integrity of *Neurexin* gene in adult heads, indeed represent a robust, reliable and convenient new approach to dissect the profile of ALS-associated features in *in vivo* based systems. In conclusion, clomipramine demonstrated strong efficacy across multiple preclinical endpoints in our *Drosophila hSOD1* models. Its ability to improve survival, modulate motor performance, mitigate inflammation, and protect genome integrity validates clomipramine as the most promising candidate identified from our *in silico* screening.

**Fig. 7:**
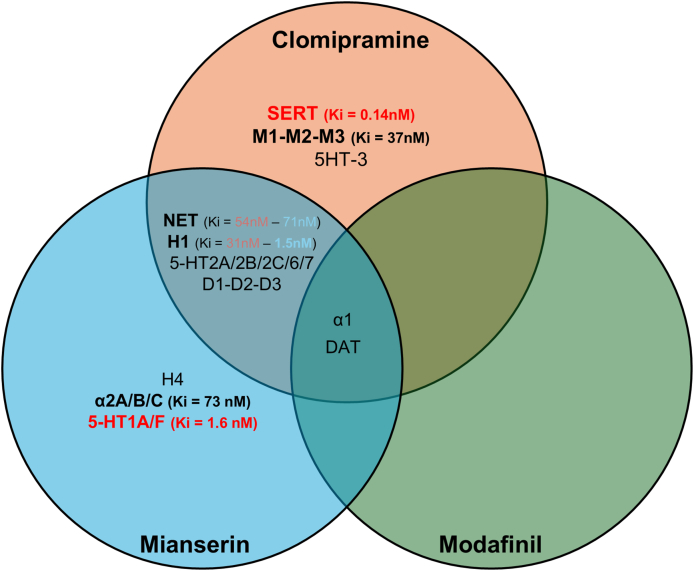
**Comparative pharmacological profile of clomipramine, mianserin and modafinil.** Venn diagram illustrates the shared and unique targets of the three compounds with correspondent Ki values (nM). Specific binding affinities are indicated as follow: red and bold ​= ​very high (Ki ​< ​10 ​nM), bold ​= ​high (10 < Ki ​> ​100 ​nM), no bold ​= ​low (Ki ​> ​100 ​nM). SERT: serotonin transporter; NET: norepinephrine transporter; DAT: dopamine transporter; H1 and H4: histamine receptors; M1, M2, M3: muscarinic receptors; 5HT-1A/1F/2A/2B/2C/3/6/7: serotonin receptors; D1/D2/D3: dopamine receptors; α1/2A/2B/2C: adrenergic receptors.

Considering that: i) the primary high-affinity-target of clomipramine is SERT (Ki ​= ​0.14 ​nM) [84]; ii) the modulation of serotoninergic pathways is increasingly recognized as a potential therapeutic route for ALS [[85], [86], [87]]; iii) SERT is structurally and functionally highly conserved from *Drosophila* (dSERT) to humans; iv) SERT inhibition has been implicated in DNA damage reduction [88]; v) the less effective mianserin and modafinil do not bind SERT (see Table 1, Table 2, Table 3, Fig. 7): we speculate that SERT engagement may likely contribute to clomipramine's therapeutic efficacy in our models. The lower affinity of clomipramine toward histamine H1 (Ki ​= ​31 ​nM), muscarinic M1-M3 (Ki ​= ​37 ​nM) receptors, and norepinephrine transporter (Ki ​= ​54 ​nM) [84,89] would likely discourage their participation in counteracting mutant hSOD1-induced toxicity.

Given these overall results, our next experimental step will be the therapeutic and mechanistic validation of these findings in the *hSOD1*^*G93A*^ALS murine model [90].

## Author contributions

**Francesco Liguori:** Conceptualization, Investigation, Formal analysis, Writing – original draft, Writing – review & editing. **Susanna Amadio:** Investigation, Formal analysis, Writing – review & editing. **Chiara Angioli:** Investigation. **Angelo Ferriero:** Investigation. **Iolanda Passaro:** Investigation. **Francesca Alberti:** Investigation. **Fiammetta Vernì:** Formal analysis, Writing – review & editing. **Cinzia Volonté:** Conceptualization, Formal analysis, Writing – original draft, Writing – review & editing, Funding acquisition.

## Ethics approval and consent to participate

Not applicable.

## Consent for publication

All authors mentioned agreed for the publication of the manuscript.

## Availability of data and materials

All data of this study are included in the manuscript.

## Funding

This study was supported by FaTALSDRUG project SAC.AD002.173.058 from National Research Council Progetti di Ricerca@CNR to C.V.

## Declaration of competing interest

The authors declare that they have no known competing financial interests or personal relationships that could have appeared to influence the work reported in this paper.

## Appendix A Supplementary data

Table S1 **Clomipramine potential ligands identified by PharmMapper analysis.** The Microsoft Excel file contains the table with the top 299 molecules identified by PharmMapper as potential ligands for clomipramine, ranked by fit score. The Filtered Rank column indicates the reordered position of these ligands after excluding those from prokaryotes, plants, and viruses (green background). Table S1
